# Acceptability and effectiveness of a monofilament, polyethylene insecticide-treated wall lining for malaria control after six months in dwellings in Vhembe District, Limpopo Province, South Africa

**DOI:** 10.1186/s12936-015-1005-8

**Published:** 2015-12-01

**Authors:** Taneshka Kruger, Mthokozisi M. Sibanda, Walter W. Focke, Maria S. Bornman, Christiaan de Jager

**Affiliations:** University of Pretoria Centre for Sustainable Malaria Control (UP CSMC), School of Health Systems and Public Health, University of Pretoria, Private Bag X323, Pretoria, 0001 South Africa; UP CSMC, Chemical Engineering, Institute of Applied Materials, University of Pretoria, Private Bag X20, Hatfield, 0028 South Africa

**Keywords:** Insecticide-treated wall lining, Polyethylene, Malaria vector control, Vhembe District, Limpopo Province, South Africa, Acceptability, Durability

## Abstract

**Background:**

South Africa uses indoor residual spraying (IRS) for vector control in its malaria control programme (MCP). Insecticide-treated wall linings (ITWLs) offer possible advantages over IRS and long-lasting, insecticide-treated nets (LLINs). This study assessed the user acceptability and perceived effectiveness, and the durability, including efficacy through bioassays, of a newly developed, monofilament polyethylene ITWL.

**Methods:**

Four ITWL formulations/treatments, two incorporated with deltamethrin and two with alpha-cypermethrin in concentrations ranging from 0.29 to 0.85 wt%, and untreated linings were randomly installed on the inner walls of traditional mud huts (n = 20) and modern brick houses (n = 20) in a community village in Vhembe District, Limpopo Province. The linings were exposed to conditions within these dwellings over 6 months. Data were collected monthly through questionnaires and entomological residual efficacy analysis of ITWL, as part of durability testing, was done bimonthly using WHO prescribed bioassays.

**Results:**

Monofilament polyethylene ITWLs were successfully installed in traditional sleeping huts and in bedrooms of modern type brick houses. ITWL remained intact throughout the entire 6 months of the study. Participants did not express any dissatisfaction towards the linings although two participants indicated the product should be fitted at a lower level for better results. User perceived effectiveness was very high with participants reporting observed mortality of mosquitoes and other nuisance insects. This perception coincided with results obtained through residual efficacy bioassays where a 100 % knockdown and mortality of mosquitoes was recorded throughout the trial period. Acceptability regarding appearance, including colour, position and attachment method, was also satisfactory with some participants citing the lining as decorative. All participants opted to keep ITWL and residual long-term efficacy will be determined annually for a further 3 years.

**Conclusions:**

The newly developed ITWLs are highly accepted amongst participants in an unsprayed section of a village in a malaria-endemic area. The perceived effectiveness that coincides with results obtained through bioassays and acceptance of the overall appearance of ITWL will be evaluated over a longer term to determine sustainability. With further developing and testing, this ITWL has the potential to become a sustainable and safer alternative vector control method.

## Background

The World Health Organization (WHO) advocates the use of indoor residual spraying (IRS) and long-lasting insecticidal nets (LLINs) as the two principal methods of malaria vector control [1]. To date vector control using IRS and LLINs has already contributed to a considerable reduction in malaria morbidity and mortality [2]. South Africa’s Malaria Control Programme (MCP) currently utilizes IRS for vector control purposes, but does not include the use of LLINs. The selection of a preferred method of vector control depends mainly on epidemiological conditions and operational settings, such as affordability, comparative efficacy and cost effectiveness of the two methods [3, 4]. Successful vector control is dependent on continued user cooperation, logistical viability and the existence of appropriate delivery systems [5]. As intervention methods, both ITNs/LLINs and IRS have demonstrated comparable levels of efficacy [6, 7], however, in spite of successful implementation both methods have their respective shortcomings. LLINs provide personal protection [8], are potentially more cost effective [9] and less difficult to utilize. However, the feeling of being confined and the personal discomfort experienced when humidity and indoor temperatures are high, affects the regular use of LLINs [5]. LLINs also only offer protection when sleeping underneath it, whilst mosquitoes can still bite people before they go to bed and are subjected to LLIN protection [8].

IRS is most effective when using dichlorodiphenyltrichloroethane (DDT), which is still used in South Africa. DDT is regarded as a persistent organic pollutant under the Stockholm Convention [10], and its use in public health is very controversial. DDT can retain its efficacy against malaria vectors for up to 12 months depending on dosage and substrate nature [11]. Alternative WHO-approved insecticides, including pyrethroids, are effective for up to 6 months [11]. The residual effect of DDT makes it more feasible to control malaria vectors in widespread rural areas [8]. DDT has not been linked scientifically to any adverse health effect but it has been suggested that it is responsible for negative health effects [12]. Research has indicated that harmful levels of DDT residue are present in the ambient air of sprayed dwellings for about 3 months after spraying has occurred. The loss of spray residue due to dust formation leads to a decrease in insecticide effectiveness on surfaces, whilst proportionally increasing the exposure potential, particularly for young children [13]. The continued success of IRS is also dependent on transcending a number of operational and financial challenges.

Safer and more sustainable alternative vector control methods should be developed to reduce the release of insecticide into the ambient air and the residual dust formation when being implemented. Insecticide-treated wall lining (ITWL), durable lining (DL) and insecticide-treated plastic sheeting (ITPS) are designed to cover interior wall surfaces, utilizing a new slow-release technology that combines the advantages of LLINs and IRS, i.e., long-lasting residual efficacy and no insecticide dusting. Similar to IRS, the intervention acts against indoor-resting vector populations [4, 14, 15], but remains active for between 3 and 5 years [5, 14] compared to IRS which has to be applied annually or even more frequently, based on the active compound [16]. The insecticide is incorporated into the lining or sheeting material during production. This is beneficial because the polymers offer a protective environment that keeps the insecticide stable for longer, whilst slowly making the insecticide available for a longer period.

A new type of ITWL has been developed at the Institute of Applied Materials (IAM) at the University of Pretoria, Pretoria, South Africa. The monofilament, polyethylene lining that can be secured to inner walls and/or ceilings of dwellings in malaria-endemic regions was produced by extruding and meshing the polyethylene directly into a net format in one step. This process is far simpler than the weaving or knitting method. The wall linings may overcome some of the limitations presented by IRS whilst offering constant indoor protection. This ITWL may contribute to lowering the cost of vector control annually and its use may reduce human exposure to insecticidal residue thereby promoting a safer and healthier environment. The loss of spray residue to dust formation would also be non-existent, allowing the insecticide to remain efficient for longer. The ITWL may therefore be a potential safer and more sustainable alternative to IRS.

In order to achieve optimum effectiveness of malaria vector interventions, user acceptance and compliance is very important. Acceptance and compliance can be sustained if the ITWL appears to benefit households by eliminating vectors and other nuisance insects and have an aesthetic value [5]. This study focused on the community acceptability and perceived effectiveness of indoor wall linings in the Vhembe District, Limpopo Province, South Africa as a potential alternative vector control strategy. The longevity of the linings installed in community dwellings, exposed to conditions within these dwellings for a period of 6 months, was also investigated.

## Methods

### Lining development

The ITWL mesh was produced on a commercial line at the Huhthamaki factory in Springs, Gauteng Province, South Africa. Four monofilament, polyethylene mesh formulations/treatments containing deltamethrin and alpha-cypermethrin actives were extruded using polyethylene as base, and the pyrethroids were introduced through masterbatches [17]. Different insecticidal treatments were tested in order to determine the most suitable formulation. Both deltamethrin and alpha-cypermethrin are listed as WHO Class III pesticides. They are deemed within the normal safety range for use during vector control and are WHO Pesticide Evaluation Schemes (WHOPES)-recommended insecticides for IRS and for mosquito nets [18]. These two pyrethroids appear to be less excito-repellent than permethrin, which is also used in LLINs. A resting mosquito would potentially be in contact with the nets for longer thus permitting a lethal dose uptake from the net surface [19]. Plain, untreated mesh was also produced for use as a negative control. The different formulations are presented in Table 1. The final mesh products were ca. 420 mm wide and 700 m in length with a mass of ca. 80–120 g/m^2^. After production all five mesh types (treated and untreated) were put through bioassays to determine efficacy. The different mesh types were stored in large polyethylene bags for about 10 months at room conditions at the Department of Chemical Engineering, University of Pretoria, before installation in community dwellings. Linings were stored after production whilst study logistics (including study site selection, negotiations with local traditional leaders and ethical approval) were finalized and the start of the malaria spray season was anticipated.

**Table 1:** Formulations for the four pyrethroid-impregnated and the one non-treated mesh

Insecticide	Active (wt%)	Lining colour
Alpha-cypermethrin (Bilag, ca. 95 % technical)	0.29	Green
Alpha-cypermethrin (Bilag, ca. 95 % technical)	0.47	Orange
Deltamethrin (Targos, ca. 98 % technical)	0.52	Brown
Deltamethrin (Targos, ca. 98 % technical)	0.85	Purple
Non treated mesh	0.00	White

The base polymer for all lining formulations was polyethylene pellets

### Selected study area and village

Malaria transmission is seasonal in South Africa from October to May and spraying for vector control occurs annually prior to the start of the malaria season. Transmission by *Anopheles funestus* and *Anopheles arabiensis* peaks during the rainy period from December to February. Rural villages in the northern and eastern parts of Vhembe District are located in a medium- to high-risk malaria area [13]. A total of 1398 malaria cases were reported in Limpopo Province from October 2012 to end March 2013 and 754 (53.93 %) of these cases were from the Vhembe District. However, the malaria incidence in neighbouring countries is a major concern.

**Fig. 1:**
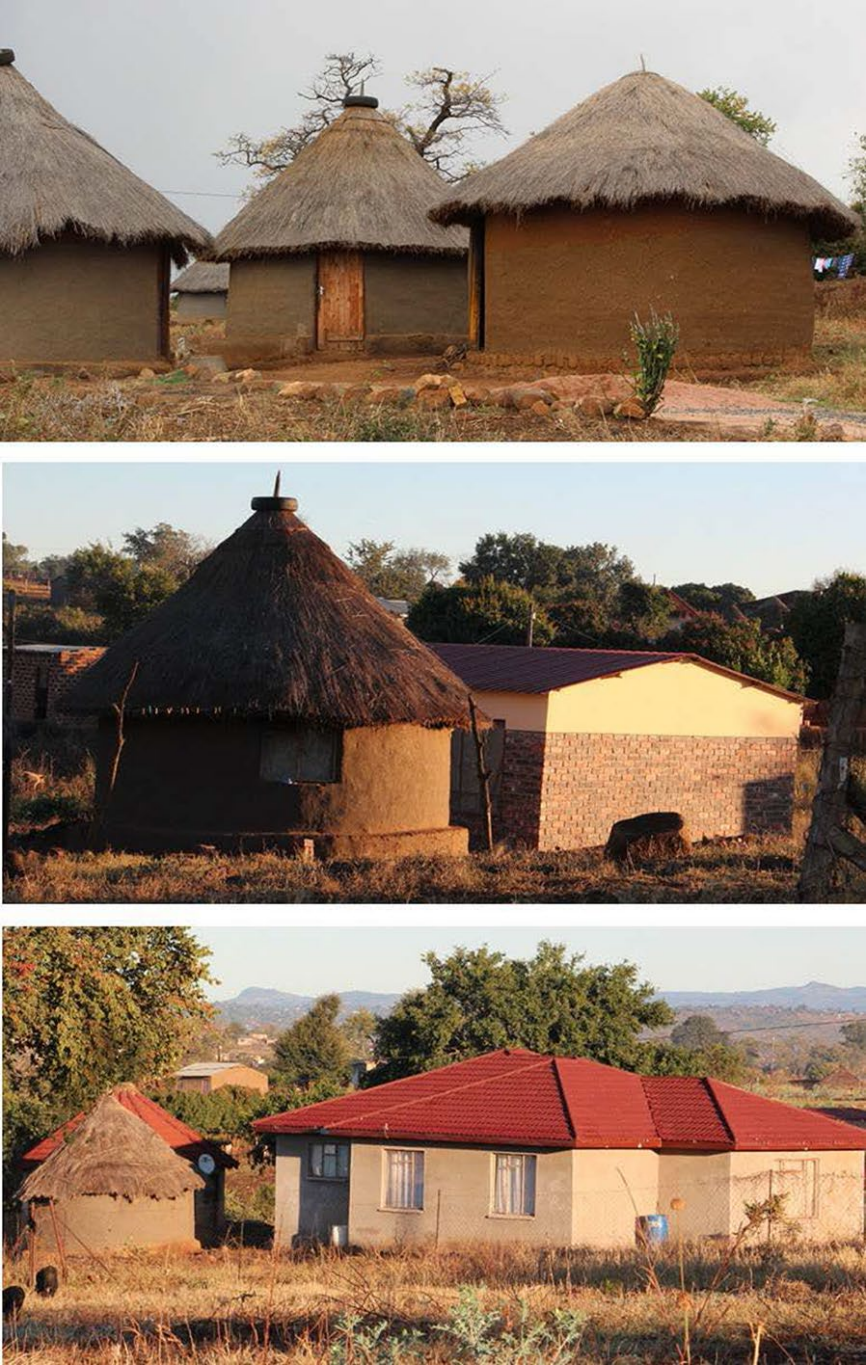
Three different Venda homesteads from Tshilivho. Traditional homestead with three traditional mud huts (*top*). More modern homesteads with an RDP house and a traditional mud hut (*middle*). Modern homesteads with a western-style house, traditional mud hut and a more modern hut with roof tiles (*bottom*). Modern homesteads do not always have traditional huts

The study was carried out from October 2012 to April 2013 in the community village of Tshilivho, which is only partially sprayed during the annual spray programme. The study focused on huts and homes in the unsprayed section of the village in order to avoid interfering with the annual IRS programme. The housing in the village (Fig. 1) consists of traditional Venda homesteads, which consist of a number of round thatch-roof huts, 3–5 m in diameter, built in a compact circular arrangement. The walls are a mixture of mud and cement, while the floors consist of mud and/or cow manure or cement [13]. The number of traditional homesteads is declining with more modern (western style) homesteads emerging. The majority of these modern homesteads are brick and cement houses built by the South African Government’s Reconstruction and Development Programme (RDP) redress policy initiative. These houses feature corrugated iron roofs. Privately built modern houses also feature corrugated iron and, in some cases, tiled roofs. More often one or two traditional mud huts still form part of the homestead, but these are mainly used as storage units or for cooking purposes, often over open fires, when it rains. Water for drinking, cooking, bathing, and washing, collected from community taps (borehole water), is usually stored on the property in large plastic containers before use. The plastic containers are often kept inside mud huts and are potential breeding habitats for mosquitoes. All participants had electricity in their homes, with the exception of one participant living in a hut.

### Informed consent and ethical considerations

Participation in the study was voluntary. The necessary permission was obtained from the Department of Health and Social Development of the Limpopo Provincial Government and local tribal authorities to conduct the study. The study was introduced to the community during two civic meetings. Written informed consent was sought from participants and this included consent to publish. Participants aged 18 years or younger (two in this study) had to sign an assent form and their legal guardians had to sign the consent form. The consent forms were translated into Tshivenda, the local language, to ensure that participants understood all aspects of the study and their rights to privacy, refusal to participate or to withdraw. Participants received an incentive in the form of pre-paid electricity vouchers to the value of R80.00 (~10USD in 2012) to compensate for the use of electricity during the installation of the linings and for the participants’ time when lining samples were collected during interviews in month 1, 2, 4, and 6. Ethics approval was obtained from the Faculty of Health Sciences Research Ethics Committee at the University of Pretoria.

### Lining installation

**Fig. 2:**
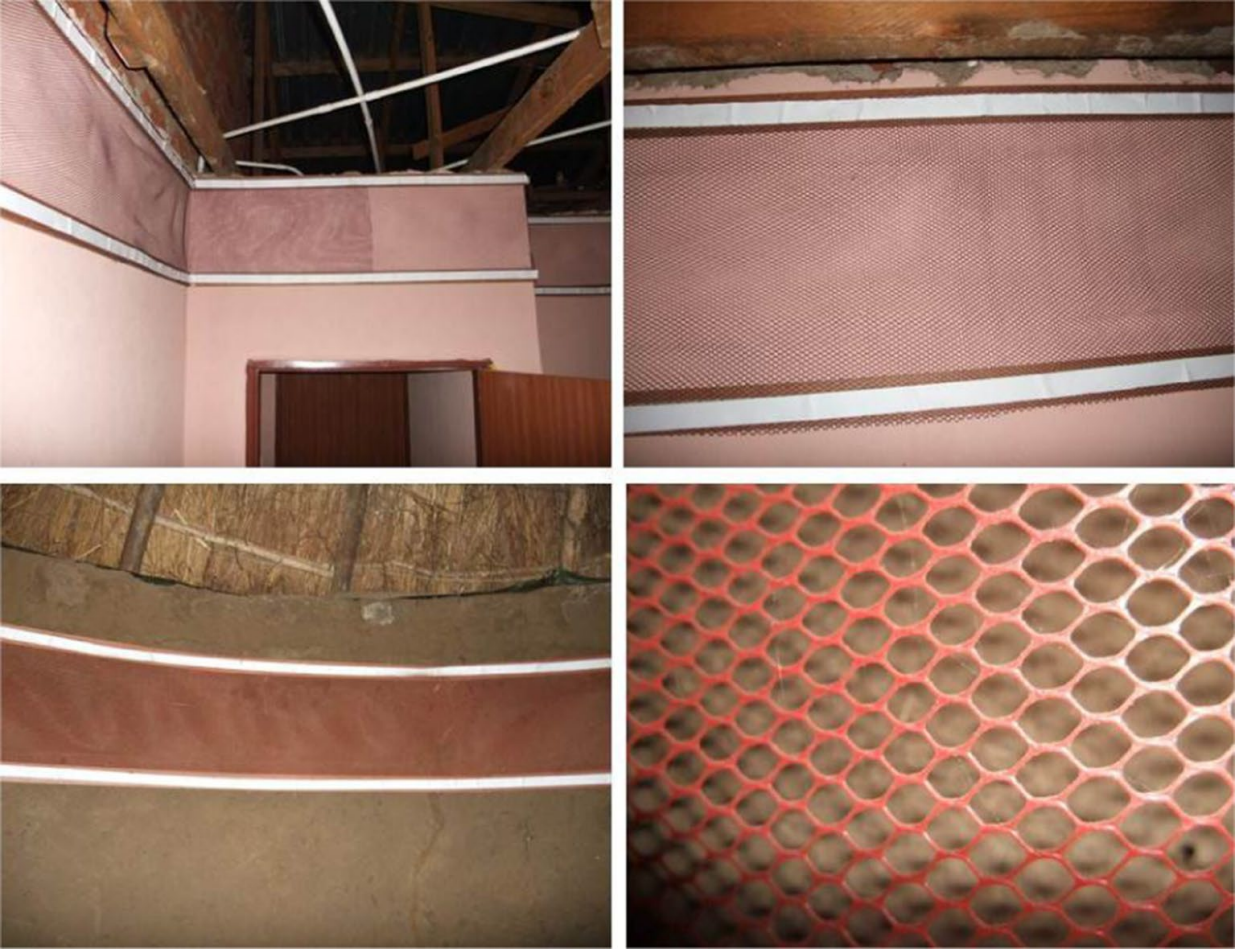
ITWL installed in community dwellings. A purple (0.85 wt% deltamethrin) lining attached with Velcro^®^ to a wooden frame mounted against the interior wall of a bedroom in a study house (*top left*). The section of overlap for efficacy sample testing is above the doorway. A close-up of the lining and attachment method (*top right*). An *orange* (0.47 wt% alpha-cypermethrin) lining installed in a sleeping hut (*bottom left*). A close-up of the extruded mesh (*bottom right*)

Forty households (20 traditional mud huts and 20 western-style houses), as determined by a biostatistician, participated in the study. The lining was installed in one bedroom in houses or in the sleeping hut (Fig. 2) of the household (henceforth termed study areas), by students from the University of Pretoria. Some of these sleeping areas served as cooking and living areas too. Each lining treatment was randomly installed in eight study sites (four huts and four houses). Installation occurred along the inside wall of the study area right below where the roof and wall met. *Anopheles arabiensis* tend to rest more often on the upper section of walls after a blood meal. Flexible wooden strips were mounted to the wall with screws at the top and about 40 cm lower down the wall to form a frame. The lining was attached to this frame by Velcro^®^ tape. The installation process was explained in detail to participants beforehand, and they were present during installation. The term participant was assigned to the person who normally slept in the study area and who was to be interviewed during scheduled visits over the 6 months. If he/she was not available then a suitable representative was identified. No instructions were given to participants on cleaning or removing wall linings. The people had to go about their daily routines without any influence from the researchers on how to ‘live’ with the new addition to their homes.

### Enrolment and questionnaire-based data collection

All data collection was conducted through interviews with questionnaires translated into *TshiVenda*. One trained *VhaVenda* field assistant conducted all interviews from informed consent of participants to the final summary interview to ensure comparable data. The assistant translated comments from participants into English. The principal investigator moderated all questionnaires to ensure they were completed in full and to query potential errors.

Prior to installation the participants completed an enrolment questionnaire that served as a baseline for future interviews. The questionnaire focused on important features of the study area: number of people (including children) that normally sleep in the study area, other purposes of the area, time spent in the area, animals and insects that enter the area, pesticide usage, methods to repel mosquitoes, spray status of the homestead, and malaria incidence in the respective households.

The acceptability, durability and the perceived effectiveness of the linings were assessed monthly. The monthly questionnaire focused on the use of pesticides for vector control, observations on the numbers of mosquitoes and insects in the study area, any damage, colour change or loosening of the linings over the month, and any observed effects on the people and animals that may have been related to the linings. The condition of the wall linings was physically evaluated during each interview.

A final, summary questionnaire was completed a week after the last monthly questionnaire: the participant’s overall perception towards the linings, attachment methods and materials, positioning of linings, the efficiency in controlling irritating insects and mosquitoes specifically; colour preferences for future products and any suggestions or complaints were assessed. Participants were encouraged to express their opinions towards the linings during all interviews.

### Laboratory-based lining residual efficacy testing

Lining durability was not only physically assessed but also subjected to entomological residual efficacy testing in the laboratory. Sample strips (100 mm wide) of each lining were collected on month 1, 2, 4, and 6 post-installation. Samples were placed in individual A4-sized, zip-lock type, plastic bags, marked and sealed. Analysis was done at the National Institute for Communicable Diseases (NICD) in Johannesburg, South Africa. Residual efficacy was determined by analysing knockdown (KD) and mortality rates of mosquitoes through WHO-recommended laboratory-scale contact or cylinder test for IRS [17, 20]. One cylinder bioassay replicate was done per lining (40 linings), with eight cylinder bioassay replicates per treatment (five treatments × eight = 40 linings). Each bioassay was carried out using 25 susceptible (to all insecticides) non-blood fed, 2–5 day old laboratory-reared female *An. arabiensis* mosquitoes KGB colony (standard strain used in testing) housed at the NICD in Johannesburg. Bioassays included blank controls where no mesh was placed in the test cylinders. Mosquitoes were exposed to the samples for 30 min. KD was recorded 30 min after the end of initial exposure and mortality was recorded 24 h after initial exposure. After exposure the mosquitoes were given sugar solution as nourishment. Efficacy was measured against the WHO efficacy criterion for IRS of a minimum of 95 % KD and a minimum of 80 % mortality post exposure.

### Data analysis

The sample size (n = 40 study areas) can be regarded as sufficient since it meets with the agreement to have at least 30 degrees of freedom for the error term in the analysis of variance (ANOVA) with response to the variable ‘slow release pyrethroid’ concentration. The study was conducted as a recurring measures factorial experiment. Quantitative data from questionnaires were entered onto an Excel spreadsheet. Acceptability criteria data (colour preference, position of the linings, etc.) and perceived efficiency were exploratory and descriptive statistics were used as required using proportions and means. Qualitative data analysis was conducted following a partially grounded theory evaluation, which allows repetitive theoretical generalizations to emerge from the data [5]. No codes were used but patterns in data were grouped together and interpreted.

## Results

### Study participants, malaria incidence and housing materials

Out of the 40 participants, 82.5 % (33/40) were female and 17.5 % (7/40) were male. Participant ages ranged from between 16 and 78 years of age at the start of the study. Fifteen per cent (6/40) of the participants, all living in huts, indicated that they had no knowledge of malaria. Only two people, both staying in huts, had ever been diagnosed with malaria: one participant’s wife (in the year 1967) and another participant’s husband (in the year 1989). One trial house (1/20) had ever been sprayed by DDT spray workers for malaria, whilst 35 % (7/20) of the study huts had been sprayed before, but all less than 6 years ago. However, not all participants had spray cards available and the information is based on what the participant could remember if they had knowledge of spray status. Two participants (2/40) possessed one untreated bed net each that had been used before. The bed nets were not used during the study.

Some important features of the housing structures that can impact on mosquito access or the effectiveness of IRS insecticides are presented in Table 2. Roof material can impact on the number of mosquitoes drawn to a dwelling. The presence of windows with glass and presence or absence of a ceiling can impact on mosquito access to dwellings. Gaps between the roofs and tops of the walls (eave gaps) that were larger than 2 cm were present in 95 % (19/20) of the traditional huts and 15 % (3/20) of the houses. The gaps allow for easy access routes in and out of houses and could potentially impact on the number of mosquitoes in the homes. Coverage of the inner walls is important due to the re-smearing of hut walls with fresh mud and cement every year or every second year.

**Table 2:** Features of the housing structures included in the study that may affect mosquito access or insecticide effectiveness

Feature	Huts (n = 20)	Houses (n = 20)
Roof material	100 % thatch roofs	90 % (18/20) corrugated iron and 10 % (2/20) tiled
Eave gaps	95 % (19/20) with opening larger than 2 cm	15 % (3/20) with opening larger than 2 cm
Ceiling	0 % with ceiling	15 % (3/20) with ceiling of standard ceiling board
Windows	55 % (11/20) with windows, all with glass, one could not open	100 % with windows, all with glass, all could open
Inner wall coverage	60 % (12/20) daub smeared (mud and cement), 25 % (5/20) lime white-washed, 10 % (2/20) plastered and one painted	40 % (8/20) painted and 60 % (12/20) plastered only

On average, 2.6 (range 1–8) people slept in a study area and 75 % (15/20) of huts and 50 % (10/20) houses had children under the age of 13 that slept there. Nineteen of 20 participants in houses indicated that they only slept in the room where the lining was to be installed versus 85 % (17/20) of participants in huts. The remaining 5 and 15 %, respectively, cooked in the study area, and this percentage changed over the study period especially in the huts over the rainy season.

Participants were asked for the time (on average) that they normally went to bed and the time that they got up in the mornings. This was done to determine the range of time when the participants would be inside the dwelling under the protection of the linings (Table 3). Majority of the people went to bed after 21:00 h and majority woke up between 06:00 and 07:00 h. The time does not indicate when they entered the sleeping areas and closed the door for the night or when they exited the area in the morning to start the day.

**Table 3:** Demographics and living habits of participants (pre-lining installation) within the study areas

Demographics and habits	Specifics	Hut	House
Participant gender	Male	25 % (5/20)	10 % (2/20)
	Female	75 % (15/20)	90 % (18/20)
Age range	<20	10 % (2/20)	N/A
	21–40	35 % (7/20)	60 % (12/20)
	41–60	40 % (8/20)	25 % (5/20)
	61–80	15 % (3/20)	15 % (3/20)
Number of people that slept in study area	1	10 % (2/20)	20 % (4/20)
	2	40 % (8/20)	40 % (8/20)
	3	35 % (7/20)	20 % (4/20)
	More than 3	15 % (3/20)	20 % (4/20)
Children in study area	<13 years of age	75 % (15/20)	50 % (10/20)
Purpose of study area	Only slept in	85 % (17/20)	95 % (19/20)
	To cook and sleep in	15 % (2/20)	5 % (1/20)
Bed time	19:00–20:00	25 % (5/20)	10 % (2/20)
	20:00–21:00	45 % (9/20)	40 % (8/20)
	After 21:00	30 % (6/20)	50 % (10/20)
Wake up time	Before 05:00	30 % (6/20)	25 % (5/20)
	05:00 and 06:00	40 % (8/20)	15 % (3/20)
	06:00 and 07:00	15 % (3/20)	50 % (10/20)
	After 07:00	15 % (3/20)	10 % (2/20)

### Installation of linings

Mounting of the frames and lining installation as described, took longer than anticipated. Challenges included hard walls that affected hole drilling for the screws to mount the frame. The reason for using flexible wooden strips was to compensate for the circular shape of the traditional huts and the ease to remove or change the lining if this was required during the study. Two to three people were needed to install the wall linings because the mesh had to be cut to fit the area’s dimensions. Furniture and containers filled with water had to be moved away from the wall, but no permanent re-arrangement of furniture occurred. All participants indicated that they were pleased with the installation process and they had no problem with the people who did the installation. Three of the 40 participants commented that the process took longer than was explained to them.

### Lining perceived effectiveness and durability over six months

One month post-installation, all ITWLs were still in position and all the participants expressed their approval of the linings. During the 1 month post-installation interview one participant, from a house, indicated that there was a bad smell for a couple of days after installation, but no negative effects (headache, nausea, etc.) were experienced. The source of the smell was not identified. No further bad smells were noted as the study continued.

Participants noted that the number, bites or irritation of mosquitoes had decreased since lining installation.

*“Net helps a lot because there are not as many mosquitoes like before. Like nets for both rooms”* (female, age 28, house with green lining).

A decrease in other nuisance insects, including cockroaches, ants, flies, in some instances, termites and summer flyers (type of month), and also spiders were noted.

*“The net is so good to us because it kills mosquitoes and other insects because every day when we sweep, we sweep many of this insects”* (female, age 21, house with orange lining).

Observations were based on the number of dead mosquitoes and insects on the floor, dead on the furniture, dead when cleaning in general, bites, and irritation. The scale of decrease (a little, more than a little or a lot) is presented in Table 4. A diagram consisting of three squares with different numbers of mosquito images per square was used to standardize responses. Similar responses to the first month were noted at the second month post-installation interview.

**Table 4:** Summary of participant responses to questions post-installation of ITWL over 6 months

	Study area	November 2012 month 1	December 2012 month 2	January 2013 month 3	February 2013 month 4	March 2013 month 5	April 2013 month 6
Decrease in mosquitoes and insects	Hut	100 % (20/20)	95 % (19/20)	100 % (20/20)	55 % (11/20)	90 % (18/20)	100 % (20/20)
	House	90 % (18/20)	100 % (20/20)	100 % (20/20)	85 % (17/20)	90 % (18/20)	100 % (20/20)
Scale of decrease: a little	Hut	N/A	11 % (2/19/)	10 % (2/20/)	9 % (1/11)	N/A	N/A
	House	N/A	5 % (1/20)	N/A	18 % (3/17)	6 % (1/18)	N/A
Scale of decrease: more than a little	Hut	5 % (1/20)	32 % (6/19/)	25 % (5/20/)	64 % (7/11)	56 % (10/18)	N/A
	House	33 % (6/18)	20 % (4/20)	30 % (6/20)	41 % (7/17)	22 % (4/18)	10 % (2/20)
Scale of decrease: a lot	Hut	95 % (19/20)	57 % (11/19)	65 % (13/20)	27 % (3/11)	44 % (8/18)	100 % (20/20)
	House	67 % (12/18)	75 % (15/20)	70 % (14/20)	82 % (7/17)	72 % (13/18)	90 % (18/20)
Rats noted in study area	Hut	15 % (3/20)	40 % (8/20)	15 % (3/20)	15 % (3/20)	5 % (1/20)	10 % (2/20)
	House	N/A	N/A	N/A	N/A	N/A	N/A

During the third month, high rainfall occurred for more than 2 weeks causing floods in parts of Mozambique and in the Kruger National Park, about 55 km from the study village. All linings were still in position and still effective according to the participants. Since the previous interviews, one participant had moved out of the hut with the lining and started using it as a cooking area (over an open wood fire).

At 4 months post-installation, participants reported that there was an increase in mosquito irritation and biting even in the study areas, possibly due to an increase in mosquito populations after the heavy rains.

*“Too much mosquito biting all over even in the room where the lining is but mosquitoes fall on the floor being dead”* (female, age 21, house with brown lining).

**Fig. 3:**
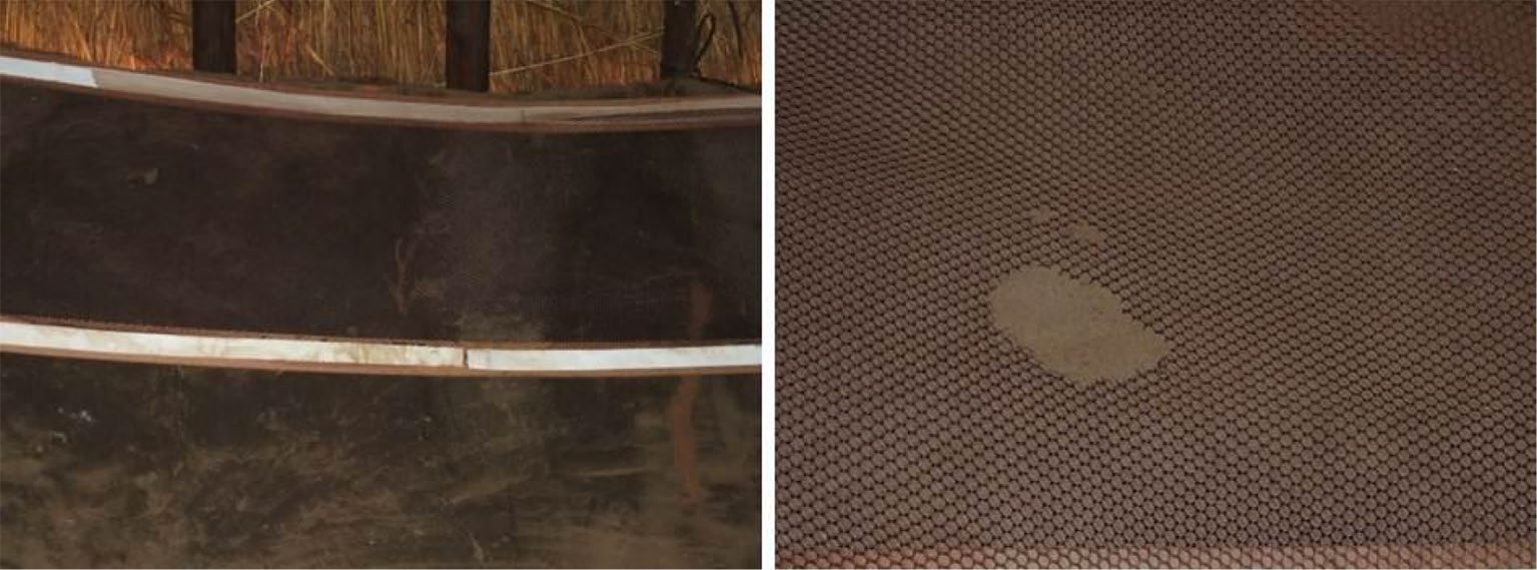
Water and rodent damage observed on a *purple* lining installed in a hut during the month four interview. The wall is damp/wet in the darker sections (*left*). Water damage in the form of brown watermarks can be seen on the Velcro^®^. A close-up of the rodent damage noted on the lining (*right*)

One participant’s cooking hut collapsed due to the rain and she started cooking over an open wood fire in her sleeping hut. Water damage was observed in her sleep hut, leaving large sections of the wall damp and parts of the lining came undone. Brown water marks on the white Velcro^®^ were visible (Fig. 3). Holes were noted in the lining and the participant mentioned that she saw rats climb on the lining (Fig. 3). Rats entered the huts of other participants (Table 4) but no other linings were damaged.

At 5 months post-installation mosquito irritation and biting was still noticed, but was lower than the previous month. One participant decided to start sleeping in a different room in her house but mosquito irritation was too high and she moved back into the study area a week later.

*“The net is helping us because mosquitoes are not as many as before when there is no lining”* (female, age 73, hut with brown lining).

After 6 months, 98 % (39/40) of the ITWLs were still in position. The lining that had come undone due to water damage did so again and the Velcro^®^ was replaced.

### Decrease in mosquito irritation and use of insecticides and repellents

**Fig. 4:**
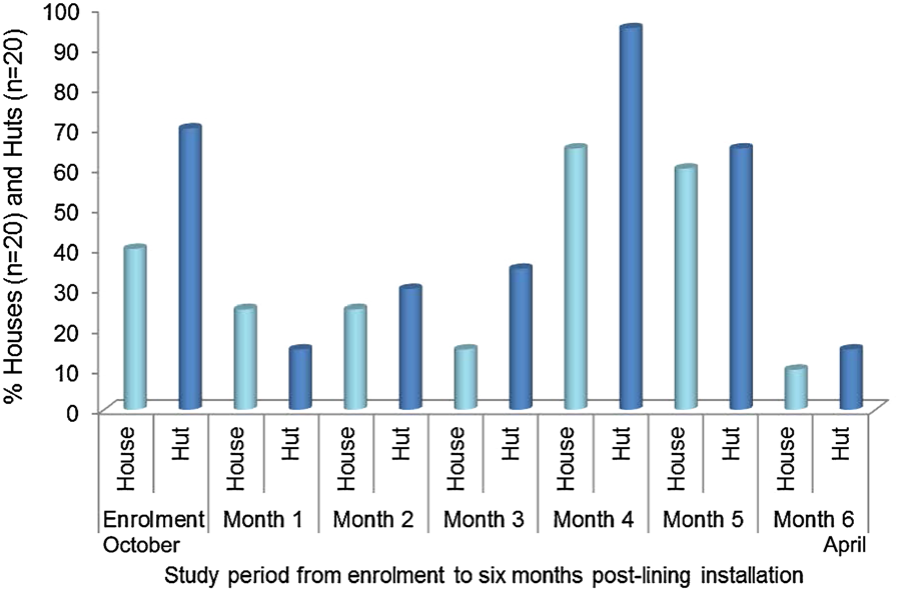
Participant perception on lining effectiveness, based on mosquito irritation whilst sleeping, over a period of 6 months. Heavy rain was experienced during month 3 and an increase in mosquito irritation was noted during month 4. At month 6 the irritation decreased noticeably

**Fig. 5:**
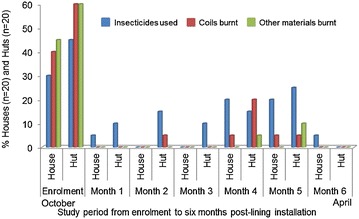
Use of insecticides and burning of mosquito coils and other materials to kill or repel irritating mosquitoes. Initial usage of the items was high at enrolment but drastically decreased. There was a slight increase in usage at month 4 after rains and a decrease thereafter. Other materials include cow dung, egg container, toilet paper and selected plant material

Over the study period, from enrolment to 6 months post-installation, a pattern was noticed in the mosquito irritation whilst sleeping (Fig. 4) compared to the use of insecticides and the burning of mosquito coils and other materials as repellents (Fig. 5). At the start of the malaria season, prior to lining installation, irritation was higher in huts where 70 % (14/20) of the participants were irritated whilst sleeping versus 40 % (8/20) of participants in houses.

Thirty per cent (6/20) of participants in houses used insecticides to kill mosquitoes and other annoying insects compared to 45 % (9/20) in huts. The burning of mosquito coils to repel mosquitoes was done in 40 % (8/20) of the houses and 60 % (12/20) of the huts, respectively. Circumstantial evidence exists that smoke is an effective insect repellent [21] and the burning of items such as dung, toilet paper, egg cartons, or plant material to produce smoke to repel mosquitoes is often practised by the *VhaVenda* people. Overall, the participants in huts burnt more of these other materials to repel mosquitoes pre-installation.

Within the first month post-installation a drop in mosquito irritation (based on perception) was noted by 25 % (5/20) of participants in houses and 15 % (3/20) in huts (Fig. 4). At the same time, decline in the use of insecticides and mosquito coils was noted (Fig. 5). An increase in mosquitoes as the season moved on is noted in the slight increase of irritation reported by the participants at 2 months post-installation. A similar trend was noted at 3 months post-installation. A drastic increase in mosquito irritation was noted at the fourth monthly interview where 65 % (13/20) of participants in houses and 95 % (19/20) in huts were irritated whilst sleeping. An increase in insecticide and mosquito coil usage was noted at this point including the burning of other materials. Irritation was still high in the fifth month but already lower than the previous month. However, a drastic decrease in irritation was noted over the last month of the study, with 10 % (2/20) of the participants in houses and 15 % (3/20) in huts indicating some irritation by mosquitoes whilst sleeping. Insecticide and coil usage was almost non-existent during the final month.

### Lining acceptability and completion of the study

None of the participants indicated a dislike of the ITWL over the study period. The final questionnaire offered participants the chance to give their overall feelings regarding the linings. All participants responded in the positive to all lining features and almost no suggestions to alter these were presented.

There was no colour change noticed in any of the linings over the trial period except for dust coverage that made white (untreated) linings appear brown. All the participants were pleased with the colour of the lining that was installed in their homes. Reasons for the positive response towards lining colour varied (Table 5) but not all participants supplied reasons. Eight of the 40 participants indicated that the linings were decorative.

**Table 5:** Reasons for positive response towards lining colour as supplied by participants

Positive aspect of colour	Huts	Houses
Decorative	20 % (4/20)	20 % (4/20)
Matches the wall colour	10 % (2/20)	10 % (2/20)
Attracts attention	10 % (2/20)	5 % (1/20)
Colour is not too bright	10 % (2/20)	15 % (3/20)
Not too visible against the wall	5 % (1/20)	N/A
No response	45 % (9/20)	50 % (10/20)

*“The net is too much good. It decorate my room and it kill mosquitoes and cockroaches”* (female, age 48, house with orange lining).

Nine colour samples were shown to the participants to determine potential future lining colour preferences. The most frequently expressed colour choices in the houses were red (13/20), green (12/20) and yellow (10/20), with the colours orange (12/20), yellow (11/20) and blue (11/20) in the huts—all bright colours. The more natural lighter (white and cream) and also darker (black, brown and purple) colours were favoured less. The *VhaVenda* people love bright colours especially in their traditional clothing and this can be noted in their colour preferences. Some participants indicated that they were happy that the lining colour was not too bright (visible). Important to note is that bright colours become darker and duller once the insecticide gets added to the mix.

Participants were in favour of the original positioning of the ITWLs (Table 6). Two participants appreciated that the lining was placed out of the reach of children.

**Table 6:** Alternative position options of lining installation

Position	Huts	Houses
Against the wall (as in study)	100 % (20/20)	100 % (20/20)
Against the ceiling	50 % (10/20)	20 % (4/20)
In front of the windows	30 % (14/20)	40 % (8/20)
Entire inside of wall	65 % (13/20)	30 % (6/20)
Under furniture	65 % (13/20)	25 % (5/20)

Participants were permitted to select more than one option

*“The net is good because it is hung up so high away of children”* (female, age 18, hut with a purple lining).

Two participants indicated a preference for the linings to be lower down the wall, whilst one wanted the lining to cover his bed.

*“The net is too much high thus why the mosquitoes are not dying and every day the number of mosquitoes is increasing”* (male, age 76, hut with a green lining).

These two participants had no children under the age of 13 that slept in the study area. Therefore, lining damage or children’s health was not a concern. Surprising though is that 65 % (13/20) participants in the traditional huts indicated that the lining could cover the entire wall, in spite of 75 % (15/20) indicating that young children stayed with them.

All participants indicated that they were pleased with the attachment method of the ITWL. A list of alternative attachment methods were presented to the participants and each was allowed to select more than one preference. The preferred alternative method was capped nails, and screws were a close second option (Table 7). Glue and staples, both more permanent methods, ranked high with participants in huts at 50 % (10/20) and 55 % (11/20), respectively. This was less in houses at 30 % (6/20) and 35 % (7/20), respectively. No suggestions for other methods were presented, but comments were offered on the method utilized in the study. Four participants (4/40) indicated that the frame covered holes after installation, and 8 % (3/40) of participants said the attachment method was decorative.

**Table 7:** Alternative lining attachment method options presented to participants

Attachment method	Huts	Houses
Glue	50 % (10/20)	30 % (6/20)
Tape	40 % (8/20)	25 % (5/20)
Staples	55 % (11/20)	35 % (7/20)
Capped nails	70 % (14/20)	55 % (11/20)
Screws	70 % (14/20)	40 % (8/20)
Rope	20 % (4/20)	20 % (4/20)
Spring rod	55 % (11/20)	40 % (8/20)

Participants were permitted to select more than one option

In the final questionnaire participants were asked if they thought the decrease in mosquitoes and other insects was due to the ITWL. The answer was a unanimous yes. A follow-on question enquired as to what happened to the number of mosquitoes and insects over time. Fifteen out of 20 (75 %) participants from huts and 85 % (17/20) from houses responded that the numbers kept on decreasing. None of the participants responded that numbers kept increasing or stayed the same. Eight out of 40 participants, 25 % (5/20) from huts and 15 % (3/20) from houses, responded that they noticed a decrease in mosquito numbers at first, then an increase for a short period, followed by a decrease again. The eight participants were requested to select options from a list to explain why they thought this had occurred. All selected that there were too many mosquitoes due to rain, and some also selected that there were usually more mosquitoes later on in the season (Table 8). No participant indicated that they thought the linings stopped working. After the trial of 6 months was completed, participants were asked if they preferred to retain the ITWL for longer.

**Table 8:** Participants’ reasoning as to why there was an initial decrease followed by an increase in mosquito and other insect numbers

Reason	Huts (n = 5)	Houses (n = 3)
Lining stopped working	100 % (5/5) disagreed	100 % (3/3) disagreed
More mosquitoes after heavy rain	100 % (5/5) agreed	100 % (3/3) agreed
Always more mosquitoes later in the season	100 % (5/5) agreed	67 % (2/3) agreed
Other	N/A	One response “When many fruits rotten they can cause many mosquitoes”

### Lining entomological efficacy over six-month period

**Fig. 6:**
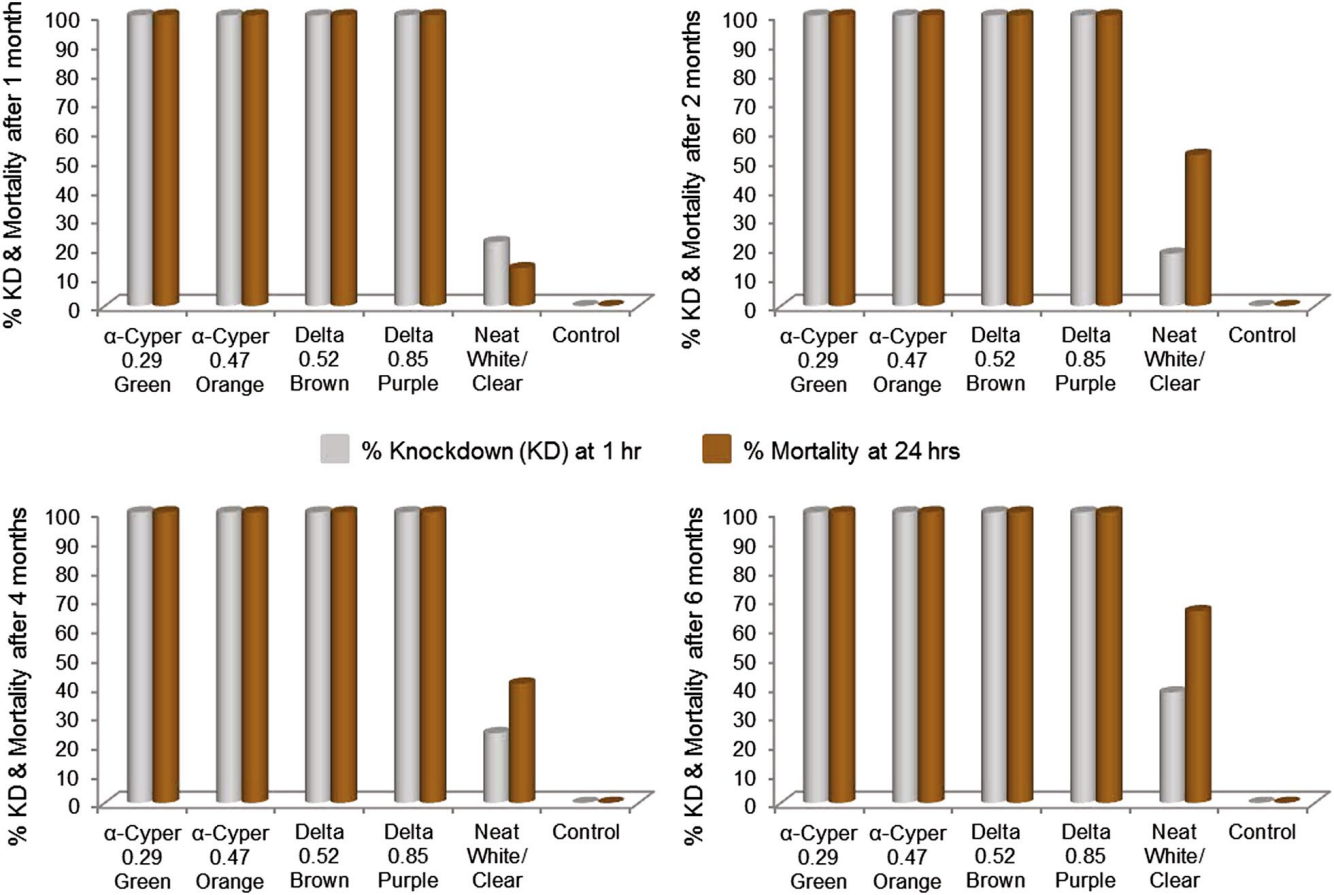
ITWL entomological residual efficacy results for samples collected over selected periods installation. The controls had no lining sample in the exposure cylinder. Untreated samples caused knockdown and mortality in laboratory mosquitoes, albeit below WHO criterion levels

Efficacy was determined as part of lining durability. The WHO effectiveness criterion is mortality exceeding 80 % after 24 h following a 30-min exposure of *An. arabiensis* mosquitoes to test samples. KD is used to indicate the bio-availability of the insecticide. All ITWL samples achieved 100 % KD and 100 % mortality right through to 6 months post-installation in study homes (Fig. 6). Samples of the water-damaged lining, soot and dirt-covered linings (especially after 6 months) still resulted in 100 % KD and 100 % mortality.

Some of the participants with untreated ITWL indicated that mosquito numbers, biting and irritation had decreased as was reported by participants with treated ITWL over the 6-month period. At first this was suspicious: participants were experiencing a placebo effect or reporting what they thought the researchers wanted to hear. However, results from the bioassays coincided with what participants perceived as a decrease in mosquitoes most likely due to the linings. KD and mortality was recorded in some of the untreated samples that should not have occurred, but this was observed at levels below WHO criteria in all the untreated samples. There exists a possibility that the untreated roll of mesh was contaminated whilst it was stored with the four treated mesh types. The initial bioassays after production resulted in no KD or mortality in the untreated mesh. The blank controls (no mesh in cylinders) resulted in no mosquito mortality during any of the post-installation efficacy analyses.

## Discussion

The aim of this study was to determine the user acceptability, perceived effectiveness and durability of the newly developed ITWL. In malaria control, the lack of effectiveness of some control interventions could potentially be a result of resistance in one of the three components required for malaria transmission, namely: parasite resistance to drugs, physiological resistance of vectors, and human resistance or non-acceptance of interventions [22]. In order for the wall lining to be considered a potential substitute for IRS, the people who live in malaria-endemic areas need to accept the linings in their homes. Without acceptance there will be no compliance to keep linings in place, therefore rendering them useless as a vector control method regardless how effective they appear in any test. The wall lining must be desirable enough for a user to want to retain it for a long period without continuous external encouragement [5].

One of the numerous potential obstacles to successful malaria control interventions is posed by the local cultural settings that are often associated with communities at risk of malaria [23]. There was high interest amongst community members once they were informed of the study. Potential participants were receptive towards the linings that were to be installed in their homes. However, in spite of the community meetings and informed consent regarding the study, a community member that originally showed interest to take part refused to sign the consent form, citing witchcraft as a concern. Cultural and religious considerations are thus very important when looking to the acceptability of new interventions in a community.

In order to sustain user compliance, the wall lining must benefit the household either through its aesthetic value or through the elimination of irritating mosquitoes and other nuisance insects [5]. Aesthetics held some value for participants and the linings were described as decorative in some instances. All participants indicated that they were content with their lining colour. Where other colours could be selected, bright colours were favoured more than darker and light colours. Participants in a study on ITPS acceptability as a vector control method in Papua New Guinea had to select a colour for future product design. The least preferred colours were darker colours because they darkened the room. Light colours were the second least favoured because they would reveal dirt more easily [16].

Two participants were pleased that the lining was out of reach of the children. The study on ITPS acceptability in Papua New Guinea highlighted the problem of children coming into contact with the sheeting that covered dwelling walls from the roof to the floor [16]. The potential for children to handle and damage the ITPS material was noted by participants, and some took precautions to prevent such damage. Similar problems were noted as potential sources of damage to LLINs [16, 24]. The concern over the possibility of side effects, specifically in small children, from the insecticide imbued in ITPS [16], was also noted. The lining size could be altered if needed. The eave gaps allow for more mosquito access and have been associated with an increased risk of malaria in some contexts [21]. The lining could be made smaller or placed to cover eave gaps that would deter mosquitoes from entering dwellings via this route, potentially lowering the risk of malaria transmission.

Cooking over open fire in unventilated circumstances in some of the huts may affect acceptability over a longer period. The exposure of the linings to smoke from these open fires leads to build-up of soot, which may not only reduce the insecticide effectiveness and durability over time but may reduce the aesthetic appeal [16] of the lining. None of the participants expressed a concern over the linings as potential fire hazards. Build-up of dirt over longer periods may also impact on aesthetics and insecticide effectiveness. People may want to remove linings and wash them to get rid of dirt (and soot). Linings covered in soot or dust was not an issue over the short term study where soot- and/or dust-covered linings retained 100 % mortality during efficacy bioassays. However, this may become an issue over a longer period. In Timor-Leste, women reported that dust on treated bed nets was a primary reason why children did not want to sleep under nets. These nets were reportedly washed every few months and in some cases every few weeks, which could severely degrade their performance [24]. In an Angolan study on urban and rural preferences of ITPS, durable lining and ITWL, dirt was one of the reasons provided as to why participants removed the materials prematurely [5].

Coverage of the inner walls is important due to the cultural tradition of re-smearing hut walls and floors each year or every second year around the festive season when the men return home from the cities to spend time with their families. This is a problem for vector control in villages where IRS does occur. Effective IRS is crippled by household members re-plastering or re-smearing walls due to stains caused after spraying occurs [8]. Traditional re-smearing of walls would become a non-factor with the ITWL, as would the washing or re-painting of walls in the more modern type houses. The linings could be removed and re-installed after re-smearing, re-painting or washing of walls. The application of IRS cannot guarantee uniform coverage unless surfaces are smooth and non-porous. The pre-treatment of the linings ensures equal distribution of insecticides regardless of the texture or surface area being covered.

Decreases reported in mosquito nuisance and biting in general, and other annoying insects post-installation suggests that entomological efficiency could be considered motivation enough for community compliance towards the continuous usage of the ITWL. Participants perceived linings as effective in spite of an increase in mosquito annoyance after heavy rains. The wall linings were perceived as killing mosquitoes, resulting in less biting and annoyance than in rooms or huts without the wall linings. In the Angolan study on preferences of ITPS, durable lining and ITWL, it was observed that user acceptability was greatly determined by the perceived entomological effectiveness of the materials to kill mosquitoes rather than their aesthetics. The participants removed the interventions as soon as they were perceived as ineffectual, which coincided with the end of the rainy season in Angola [5]. If other insects are killed by the wall lining then users may keep the intervention in position even during times of low mosquito numbers. Lack of mosquito nuisance is often provided as a reason for non-use of bed nets [25].

Dwellings constructed out of mud and thatch, or similar ‘natural’ materials, attract more mosquitoes than houses constructed from brick, cement, asbestos, and metal components [8]. On average, participants in traditional mud huts did indicate higher mosquito annoyance than those living in more western-style or RDP houses. This observation was supported by the usage of more insecticides to repel or kill mosquitoes and other annoying insects. The burning of mosquito coils and other items to repel insects was also higher in the traditional huts. During a nationwide survey in Malawi, 64 % of households reported burning leaves, wood or dung to produce smoke to repel mosquitoes. Smoke may influence mosquito biting behaviour by masking human odours and carbon dioxide used for short-range host location, lowering humidity, which affects mosquito chemoreceptors that are more responsive in the presence of moisture, and acting as irritants, repellents or insecticides because of various organic compounds within certain plant smokes. The incomplete combustion of these materials forms a smoke mixture of particles, chemicals and gases that could be potentially hazardous to health [21]. Therefore, the use of the linings would promote a healthier home environment due to less usage of insecticides and the burning of mosquito coils and other materials. This in turn could impact positively on a user’s finances. The *VhaVenda* people appear to be moving away from traditional homesteads as more brick and cement homes are being built. However, villages further north are still very traditional.

Participant responses during interviews may have been influenced by social appeal bias [16]. Participants appeared to be truly appreciative and accepting of the linings. In spite of constant encouragement to be truthful in their responses to questions, participants may have supplied answers that they believed the researcher wanted to hear. All the participants indicated that they would recommend the linings for future use against mosquitoes and other insects to other members of their village or people from other villages. This is a clear indication of acceptance of the ITWL by the participants and an important step towards the feasibility of testing these linings further as an alternative method for vector control. What is important with all malaria interventions is that the communities or intervention users need to be educated on malaria, the various methods of controlling the disease, the purpose of ITWL and why it is potentially safer and more sustainable than IRS. This should greatly influence the compliance of users to retain ITWL for longer, making them more effective.

### Study limitations and recommendations

These types of preliminary studies are important to determine user preferences towards a new product’s development and design [5]. There are a couple of issues and recommendations to consider in future studies. The ITWLs were installed in a section of the village that is not readily affected by malaria. The linings demonstrated their efficiency against mosquitoes in general along with other annoying insects, and they were well received by participants. However, in spite of this, the overall purpose of such an intervention is to effectively prevent malaria transmission and not look decorative in a user’s home. Before WHOPES evaluation is considered, further testing should be done with the functionality of the lining in mind. Experimental hut trials should occur to determine efficacy with regard to preventing mosquitoes from entering huts and having access to a blood meal. This will present a better understanding of ITWL transmission prevention capabilities. Epidemiological and entomological surveys should be done.

Alternative methods for faster and easier installation should be researched. This will enable community members to remove and re-install ITWL themselves as needed, especially when re-smearing of huts, painting of walls or cleaning of linings occurs. An economic feasibility study should be completed to determine the production and implementation costs and to compare the annual cost against that of IRS as vector control programmes.

The lining acceptability and durability should be looked at over a longer period. Factors such as dirt build-up over time will alter the aesthetics that in turn may affect lining acceptance and user compliance. The effectiveness over a longer period should be analysed to determine how long ITWL remains functional in the homes of users. At the end of the study participants were presented with the option to retain the linings in their homes for longer and all participants opted to do so. Permission was obtained to collect samples annually for at least 3 years for further efficacy testing (until end 2015). The effect of washing on ITWL efficacy should also be determined, especially over the long-term period.

Pyrethroids are currently the only insecticides approved by the WHO for use on/in ITNs or LLINs and also ITWL. However, vector pyrethroid resistance is of major concern and alternative insecticides should be tested for use in ITWL. Researchers at the University of Pretoria are already investigating alternative insecticides to use in ITWL.

## Conclusions

Insecticide-treated wall lining is still a relatively new vector control method. The newly developed monofilament polyethylene ITWL that was assessed during this small-scale field trial over a 6-month period displayed great potential. User compliance was 100 % from the start of the study throughout the 6 months post-installation. The participants accepted the ITWL in their homes and appreciated the overall appearance and colour options, the positioning and attachment method. The perception of entomological efficiency contributed to this acceptance. Participants were impressed with the ITWL and opted to keep them for at least a further 3 years. This will allow for long-term durability and efficacy assessment. The noticeable decrease in the use of insecticides and the burning of mosquito coils and other materials within the homes is an added health bonus. The positive study results indicate that further evaluation of the non-finished product is warranted. With additional testing and eventual submission for WHOPES evaluation, this ITWL could potentially become a safer and more sustainable alternative vector control method.
